# Examination of the relationship between toilet behaviors and recurrent urinary system infection in women

**DOI:** 10.1590/1806-9282.20250836

**Published:** 2025-12-15

**Authors:** Yeliz Culha, Ezgi Seyhan Ak, Pelin Akyurek, Kahraman Aksoy, Mehmet Gokhan Culha, Alper Otunctemur

**Affiliations:** 1 Istanbul University-Cerrahpasa, Florence Nightingale Faculty of Nursing, Department of Fundamental Nursing – Istanbul, Turkey.; 2 Istanbul University-Cerrahpasa, Florence Nightingale Faculty of Nursing, Department of Surgical Diseases Nursing – Istanbul, Turkey.; 3 Health Sciences University, Prof. Dr. Cemil Taşçıoğlu City Hospital, Urology Clinic – Istanbul, Turkey.

**Keywords:** Urinary tract infections, Recurrence, Behavior, Women, Toilet training

## Abstract

**OBJECTIVE:**

This research sought to examine the relationship between toilet behaviors and recurrent urinary tract infections in women.

**METHODS:**

This descriptive correlational study was conducted with female patients with recurrent urinary tract infections who applied to the urology outpatient clinic of a city hospital in Istanbul between March 2023 and April 2024. Data were collected using the Patient Information Form and Toileting Behavior-Women's Elimination Behaviors Scale.

**RESULTS:**

The total mean score of the Toileting Behavior-Women's Elimination Behaviors Scale was found to be 49.79±9.98 in the patients. When the total mean scores of the Toileting Behavior-Women's Elimination Behaviors Scale were examined according to the individual characteristics of the patients, it was found that there was a statistical difference in the mean score of the Toileting Behavior-Women's Elimination Behaviors Scale according to marital status, education level, incontinence status, preferred toilet position inside, and outside the home (p<0.05) and that there was a statistical relationship between age and the mean score of the Toileting Behavior-Women's Elimination Behaviors Scale (p<0.05).

**CONCLUSION:**

Increasing age had a negative effect on early voiding and straining behaviors, and the presence of incontinence had a negative effect on early voiding and general toilet behavior in women with recurrent urinary tract infections.

## INTRODUCTION

Recurrent urinary tract infections (UTIs) are defined as at least three UTIs per year or two within 6 months, including both cystitis and pyelonephritis^1^. They are common among women of all ages and socioeconomic backgrounds and are a major cause of antibiotic use, leading to significant healthcare costs^2–5^.

While pharmacological treatments are standard, lifestyle changes—like proper hygiene, urinating before and after intercourse, avoiding jacuzzis, and increasing fluid intake—are believed to help prevent recurrence^4,6^. UTIs can impair women's daily lives, prompting many to avoid public places without toilet access, delay urination, or use pads^7,8^.

Toilet behaviors—encompassing place, time, position, and method of urination—play a crucial role in bladder health^7,9,10^. Some habits, such as premature or delayed voiding, straining, and altering the usual sitting position, can negatively affect bladder function over time^10^. A cross-sectional study linked overactive bladder symptoms to early and delayed voiding, difficulty urinating, and positional preferences^11^. Limited toilet access at work may increase lower urinary tract symptoms (LUTS)^10,12^.

Women often squat in public toilets for hygiene, but this may hinder pelvic relaxation and bladder emptying, contributing to incontinence^10,13–15^. Healthy toileting involves sitting comfortably, leaning forward, relaxing pelvic muscles, and avoiding straining^10,16–18^.

To address urinary voiding issues common among women, behavioral interventions are needed. Nurses can promote awareness and behavioral change to prevent UTIs and improve bladder health^7^. Identifying the link between toilet behavior and recurrent UTIs is key for developing educational programs. Although prior studies note environmental and behavioral factors affecting toileting^12,19,20^, none have explored their relationship with recurrent UTIs. This study aimed to examine that relationship.

Research hypothesis

H_1_: There is a significant relationship between unhealthy toileting behaviors (TBs) (e.g., delaying urges, changing position, incomplete evacuation, and excessive effort for hygiene) and recurrent UTIs in women.

H_0_: There is no significant relationship between TBs and recurrent UTIs in women.

## METHODS

### Study design and participants

This descriptive, correlational study was conducted from March 2023 to April 2024 in a urology outpatient clinic of a city hospital in Istanbul. Using simple random sampling, 81 women with recurrent UTIs who met the inclusion criteria and gave informed consent were included. Recurrent UTIs were defined as UTIs diagnosed by urine culture at least three times per year or two recurring uncomplicated and/or complicated UTIs within the last 6 months. Therefore, the inclusion criteria were aged 18 years or older and experiencing at least three UTIs per year or two UTIs per 6 months. Individuals with communication disorders were excluded from the study.

### Data collection tools

Data were gathered through the Patient Information Form and the TB–Women's Elimination Behavior Scale (TB-WEB).

The Patient Information Form developed based on literature, covered age, height, weight, marital status, smoking, alcohol use, pregnancy history, urinary symptoms, and toilet position preferences^20–22^.

The TB-WEB Scale, created by Wang and Palmer and adapted to Turkish by Seyhan Ak et al.^7^, assesses behaviors related to bladder emptying. It includes five subscales: place preference (4 items), early voiding (5), delayed voiding (3), straining (4), and position preference (2). Items are rated on a five-point Likert scale; higher scores reflect more negative TBs. The scale had a Cronbach's alpha of 0.81 in Seyhan Ak et al.'s study^7^ and 0.85 in this one.

### Data collection

Data were collected through face-to-face interviews in the clinic. Participants completed the forms and the TB-WEB Scale.

### Data analysis

Data were analyzed using SPSS 21.0. Kolmogorov-Smirnov tests assessed normality. T-tests, chi-square tests, ANOVA, post-hoc Tukey test, and Pearson correlation were used, with significance at p<0.05.

### Ethical considerations

Ethics approval (Number: 48670701) was obtained. Participants were informed about the study's purpose and their rights, and consented voluntarily.

## RESULTS

When the characteristics of the patients included in the study were examined, it was found that the mean age was 50.04±9.85 years, 71.6% were married, 46.9% were primary school graduates, 91.4% were not working, 58% did not smoke, 91.4% did not drink alcohol, 60.5% of the patients had chronic diseases, 38.42% of those with chronic diseases had heart disease, and 54.3% did not have urinary incontinence.

A total of 49.4% of the patients preferred sitting as their toilet position at home, and 46.9% preferred sitting as their toilet position outside the home.

The mean total score of the TB-WEB Scale was 49.79±9.98, the mean score of the preferred place for voiding subdimension was 13.62±3.13, the mean score of the premature voiding subdimension was 13.99±5.44, the mean score of the delayed voiding subdimension was 6.72±2.51, the mean score of the straining in voiding subdimension was 10.04±3.97, and the mean score of the position preference for voiding subdimension was 5.43±2.12 (Table 1).

**Table 1:** Patients’ elimination behavior score mean.

Scale	Mean	SD	Min	Max
Place preference for voiding	13.62	3.13	6	19
Premature voiding	13.99	5.44	5	22
Delayed voiding	6.72	2.51	3	12
Straining voiding	10.04	3.97	4	16
Position preference for voiding	5.43	2.12	2	8
Total	49.79	9.98	25	65

SD: standard deviation.

The analysis of the TB-WEB Scale scores revealed significant differences based on marital status, education, incontinence status, and preferred toilet position (p<0.05). In a comparison of outdoor toilet preferences, the place preference for voiding score of women who squatted was found to be higher than that of women who sat (p=0.006). The "Position preference for voiding" subscale score of women who were semistanding was higher than that of women who sitting position and squat position (p<0.001) (Table 2). A statistical relationship was also found between age and the TB-WEB Scale score (p<0.05) (Table 3).

**Table 2:** Toilet behavior-Women's Elimination Behavior Scale mean scores according to patients’ ındividual characteristics.

Characteristics		Toilet behavior-Women's Elimination Behavior Scale					
		Place preference for voiding	Premature voiding	Delayed voiding	Strainig voiding	Position preference for voiding	Total
Marital status	Married	13.90±3.05	14.50±4.92	7.34±2.38	10.41±3.41	5.72±1.76	51.88±8.60
	Single	12.91±3.27	12.70±6.52	5.13±2.12	9.09±5.10	4.70±2.74	44.52±11.40
	t; p	1.242; 0.204	1.353; 0.180	3.890; <0.001	1.362; 0.177	1.150; 0.259	**3.155; 0.002**
Educational status	Primary school	12.58±3.67	15.87±3.52	6.66±1.76	11.34±3.73	4.45±2.51	50.89±10.52
	Middle school	16.00±0.12	17.00±0.56	7.00±0.34	4.00±0.12	6.00±0.61	50.00±2.50
	High school	14.04±2.82	9.35±6.83	6.61±1.75	8.96±4.57	6.74±1.39	45.70±10.49
	University	14.88±.03	15.44±3.44	6.94±4.61	10.00±1.86	5.75±0.45	53.00±7.54
	t; p	3.466; 0.200	10.754; <0.001	6.506; 0.972	**5.941; 0.001**	7.276; <0.001	2.079; 0.110
Incontinence	Yes	13.78±2.72	15.03±5.16	7.03±2.80	12.41±2.43	5.43±1.94	53.68±6.75
	No	13.48±3.46	13.11±5.76	6.45±2.23	8.05±3.94	5.43±2.28	46.52±11.10
	t; p	0.437; 0.663	**1.591; 0.001**	1.054; 0.309	**5.854; <0.001**	0.001; 0.999	**3.422; 0.001**
Preferred toilet position at home	Sitting	13.76±3.33	14.26±5.80	6.80±2.51	10.19±4.22	5.80±1.96	50.80±10.38
	Semi standing	12.73±1.009	12.27±1.009	6.18±2.52	9.09±1.51	3.09±1.51	43.36±0.51
	Squat	13.62±3.13	13.99±5.44	6.72±2.51	10.04±3.97	5.43±2.12	49.79±9.98
	t; p	1.030; 0.313	1.268; 0.263	0.575; 0.450	0.719; 0.399	**19.130; <0.001**	**5.581; 0.021**
Preferred toilet position outside home	Sitting	12.63±3.69	16.21±5.93	7.13±2.68	10.92±4.46	5.03±2.15	51.92±11.99
	Semi standing	13.50±1.56	10.50±5.71	7.50±0.52	8.00±4.15	8.00±0.37	47.50±7.83
	Squat	14.97±2.37	12.76±2.95	5.79±.62	9.86±2.77	4.72±1.62	48.10±7.40
	t; p	**5.053; 0.009**	7.967; 0.001	3.361; 0.040	2.945; 0.058	**18.022; 0.001**	1.677; 0.194

t: Independent-sample t-test, p<0.05. Statistically significant values are presented in bold (p<0.05).

**Table 3:** Relationship between patients’ characteristics and elimination behaviors correlations.

Characteristics		TB-place preference for voiding	TB-premature voiding	TB-delayed voiding	TB-straining voiding	TB- position preference for voiding	TB-total
Age	R	0.004	0.219*	-0.243*	0.317**	-0.076	0.170
	P	0.971	0.049	0.029	0.004	0.501	0.128

r: Pearson correlation. TB: toileting behavior.

* p<0.05,

** p<0.01.

## DISCUSSION

Toilet behaviors are key to women's bladder health^9,10^. This study identified such behaviors in women with recurrent UTIs and their links to individual factors.

Most women prefer sitting at home, while squatting is more common outside due to hygiene concerns^11,23,24^. In this study, sitting remained the most common position both at home (49.4%) and outside (46.9%). Newman et al.^10^ similarly found that about half of women sit outside the home. Other studies show that 75% of women worry about public toilet cleanliness, which drives squatting^13,16^.

Hygiene concerns strongly shape TB^20^. Hygiene concerns represent a critical factor shaping women's TBs, particularly in public restroom environments. Our findings align with the existing literature demonstrating that women frequently compromise optimal voiding practices due to perceived hygiene risks in shared sanitation facilities. Reynolds et al.^12^ found that women frequently engage in unhealthy behaviors in public restrooms, increasing bladder symptoms. In this study, negative toilet habits were prevalent, often influenced by comfort, hygiene, and safety concerns. Socioeconomic and occupational factors significantly influence TBs and may contribute to the risk of recurrent UTIs. Women with limited access to clean and private restrooms, such as those in low-income or demanding occupations (e.g., healthcare workers, and factory employees), often adopt unhealthy voiding habits like delayed urination or squatting to avoid unsanitary conditions. These behaviors can lead to incomplete bladder emptying, increased residual urine, and higher UTI risk. Additionally, lower education levels and economic constraints may limit awareness of proper hygiene practices or preventive measures^21,23^.

Early voiding—urinating without need—can reduce bladder capacity and function^25^. The literature reports that nearly half of elderly women^14^ and 81% of female physicians and medical students^20^ urinate before leaving home. Wu, Xue, and Palmer^26^ also found many women void without urgency. In this study, early voiding was the most common unhealthy behavior. Since frequent urination and urgency are symptoms of UTIs^2,3^, this finding was expected. The observed association between incontinence and early voiding behaviors may represent an adaptive strategy to prevent urinary accidents rather than a direct contributor to UTI pathogenesis, suggesting these findings should be interpreted within the context of symptom management rather than causal risk factors. While early voiding was more prevalent among incontinent women in our cohort (15.03±5.16 vs. 13.11±5.76, p=0.001), this likely reflects a compensatory mechanism for bladder control rather than an independent UTI risk factor.

The "preferred place for voiding" score suggested that discomfort with public toilets shapes TB. Many studies support women's reluctance to use public restrooms due to cleanliness concerns^11,12,16,23^. For women with recurrent UTIs, avoiding such places may reflect health protection efforts, as poor hygiene can increase infection risk^27^.

Delayed voiding is common among women with demanding jobs or hygiene worries^12,13,15^. Both premature and delayed voiding are linked to LUTS and lower quality of life^20,22,28^. For instance, 53.6% of nurses^24^ and 87% of female physicians^20^ delay urination. In this study, delayed and straining behaviors were less frequent, possibly because most participants were not employed.

Urinary incontinence, though common with age, should not be viewed solely as a normal aging symptom. It also impacts toilet behavior^22^. For women with urge incontinence, early voiding may be a coping mechanism to avoid accidents^10,14^. Delayed voiding and position changes are more common among younger women, while unhealthy behaviors increase with age^10,22^. In this study, age, marital status, education, incontinence, and toilet position preferences all influenced TBs.

Squatting is often used to avoid infection, but may impair bladder emptying^11,13,23^. Position preferences vary by culture and health^9^. Though squatting helps increase intra-abdominal pressure and may support bladder emptying, studies found no difference in flow between sitting and squatting^11,29^. However, squatting may prevent full muscle relaxation, increasing residual urine and infection risk^9,30^.

### Study limitations

This study employed a descriptive correlational design, which inherently limits the ability to infer causality between toilet behaviors and recurrent UTIs (rUTIs). Although a significant association between certain toileting habits and rUTIs was observed, the temporal sequence of events cannot be confirmed. Therefore, it remains unclear whether the behaviors preceded or followed the onset of recurrent infections. Future research using longitudinal or case-control designs would be more suitable to explore potential causal relationships and the directionality of these associations.

Additionally, the absence of a control group (i.e., women without a history of rUTIs) restricts the comparative interpretation of our findings. Without a non-rUTI group for reference, it is difficult to determine whether the identified TBs are specific risk factors for rUTIs or common practices among women more generally. Inclusion of a control group in future studies would enhance the clinical relevance and generalizability of results.

## CONCLUSION

This study revealed that increasing age had a negative effect on early voiding and straining behaviors in women with recurrent UTIs and that the presence of incontinence had a negative effect on early voiding and general toilet behavior. Identifying modifiable toilet behavior factors in women with recurrent UTIs and organizing awareness-raising training programs to evaluate the effects of these behaviors on health may be important components of the management of UTIs. It is recommended that studies be planned to examine the effects of different demographic characteristics on toilet behaviors with larger sample groups.

## Data Availability

The datasets generated and/or analyzed during the current study are available from the corresponding author upon reasonable request.
